# Removal of Seven Inches of Shaft of the Fibula—Recovery of the Patient—Description of a New Instrument for Facilitating the Passage of the Chain-Saw under the Bone

**Published:** 1860-01

**Authors:** E. S. Cooper

**Affiliations:** Professor of Anatomy and Surgery in the University of the Pacific, San Fraucisco


### ARTICLE 3.

### REMOVAL OF SEVEN INCHES OF SHAFT OF THE FIBULA—RECOVERY OF THE PATIENT—DESCRIPTION OF A NEW INSTRUMENT FOR FACILITATING THE PASSAGE OF THE CHAIN-SAW UNDER THE BONE.

BY E. S. COOPER, A. M. M. D.,

Professor of Anatomy and Surgery in the University of the Pacific, San Frauclsco.

*Case.*—Mr. A. A. aged 35 years was admitted into the Pacific Clinical Infirmary, June, 1857, in consequence of long standing ulcerations and sinus openings on the outer part of the right leg. On introducing the probe through the small sinuses leading to the bone, the fibula was found in a carious condition nearly all along its shaft. The patient, a laboring man, had been entirely unable to work for many months.

I decided at once to exsect the diseased bone.

*Operation.*—The patient, being placed upon a solid table on his left side, and chloroform being administered, I made an incision nine inches long in the direction of the shaft of the fibula down to the bone. The periosteum was found thickened though otherwise healthy, save in the numerous perforations through it corresponding to the sinuses communicating with the surface. A slit was therefore made through it almost as long as the incision through the external parts, when it was readily pushed aside, leaving the bone exposed.

An instrument was then used which I had invented on purpose to facilitate the passage of the chain-saw under the bone and which I have named the chain-saw conductor.

It was made as follows: A piece of steel wrought into the shape of a needle, but about seven inches long, and thick accordingly, with a straight shaft, but curved very much at the point. An eyelet hole in the perforating extremity received the ligature which was used in drawing the saw through. The point of the instrument being thrust through the tissue very close to the bone, appeared on the opposite side of the puncture conveying the ligature which was secured, and the conductor withdrawn.

The instrument made a sufficiently large opening to admit the passage of the chain-saw with ease, as well as a piece of chamois leather, which was conveyed along with and between it and the soft parts for their better protection againgst laceration by the saw. This being arranged, there was nothing left but to divide the bone, which was done in the same manner, both above and below, seven inches apart, when the intervening section of bone was taken away.

A roll of lint was then applied in the wound, a roller tightly over the leg and foot, an evaporating lotion of one part of alcohol, to ten of water, was used to keep the dressing constantly wet for the first five days, and spirits of mindererus freely administered.

At the end of this period the cold applications were discontinued, the roller removed and poultices applied to the wound.

No burrowing matter ensued, the wound was free from inflammation during convalescence, and in three months after the operation, the patient was enjoying comparatively good health with the leg almost as useful as ever, the bone having been reproduced, or some solid substance answering as well.

The lint was permitted to remain in the wound for two weeks, when it was removed.

*Remarks.*—I am aware that the plan of leaving a piece of lint or any other foreign body in a wound of this kind, is contrary to the practice of surgeons of this day, and that it would by many be considered very reprehensible, because it is the wish of surgeons generally to have such wounds heal as nearly as possible by first intention, whereas this method most effectually prevents it.

I am convinced however, by experience that it is the true method of conducting the after-treatment of these cases because my success in the cure of such patients since I adopted it, has been much greater than before.

I deny, that atmosphere admitted into wounded bones or joints is in any way injurious, so there is no objection to having the wound open on that account, and a roll of lint pressed tightly against the cut surface of the soft parts by means of a roller applied over the limb, not only prevents secondary hemorrage from the numerous small arterial branches which are frequently wounded in these operations, but it is the means also of condensing the tissues on the cut surface and thereby interrupting the passage of purulent matter from the wound into the surrounding structures; but above all, it keeps the wound freely open for the escape of purulent matter, or even any spicula of bone which may by exfoliation subsequent to the operation, require a free opening for its removal.

In many of these cases where a large section of one of the long bones has been removed, and the reproduced tissue occupying its place is slow in hardening, I use accupuncture needles, or a small drill, by which numerous holes are made into this substance, which is often found at the expiration of three months, of less than cartiligenous hardness. But in this state I have frequently been surprised and highly gratified at the rapidity with which hardening would take place, after the drilling was begun.

The idea of this practice occurred to me in consequence of the ingenious experiments upon animals for the cure of pseudarthrosis, made by Prof. D. Brainard of Rush Medical College, some years since, in which the practicability of hardening the callus connecting the extremities of bones by a similar practice was fully demonstrated, and which is destined to exercise considerable influence upon the practice of surgeons, seeing that the operator need not hesitate to remove diseased bone since he has learned the mode of quickening nature's efforts at reproduction, when she appears either too slow in her work or stops altogether.

It becomes a question with me whether bone could not frequently be produced, and the tissues always hardened by this process, even in parts where no bone had existed before.

Nearly all the tissues tend to bone as age advances; and rheumatic inflammation, and injuries often produce ossification of ligaments.

As I have already published my views at length upon this subject, I will add no more to this communication.

